# Correlation of mild cognitive impairment with the thickness of retinal nerve fiber layer and serum indicators in type 2 diabetic patients

**DOI:** 10.3389/fendo.2023.1299206

**Published:** 2024-01-08

**Authors:** Renshi Li, Fengjie Zheng, Peichen Xu, Li Lv, Yapeng Mu, Xianghua Zhuang, Shihong Chen

**Affiliations:** Department of Endocrinology and Metabolism, Multidisciplinary Innovation Center for Nephrology of the Second Hospital of Shandong University, The Second Hospital of Shandong University, Ji’nan, Shandong, China

**Keywords:** type 2 diabetes mellitus, the thickness of retinal nerve fiber layer, interleukin-18, irisin, carboxymethyl lysine, receptors for AGEs, mild cognitive impairment

## Abstract

**Background:**

Cognitive Impairment arising from type 2 diabetes mellitus (T2DM) has garnered significant attention in recent times. However, there are few studies on the identification and diagnosis of markers of cognitive impairment. Notably, alterations in the Retinal Nerve Fiber Layer’s (RNFL) thickness can potentially serve as an indicative measure of central nervous system changes. Further investigations have indicated that the decline in cognitive function within T2DM patients is intricately linked to persistent systemic inflammation and the accumulation of advanced glycosylation end products. Comprehensive studies are warranted to unveil these complex associations.

**Objective:**

This study aims to explore the potential of utilizing the RNFL thickness and serological concentrations of IL-18, irisin, CML, and RAGE as diagnostic indicators for Mild Cognitive Impairment (MCI) among individuals with T2DM.

**Methods:**

The thickness of RNFL were determined in all patients and controls using optical coherence tomography (OCT). The serum levels of IL-18, irisin, CML and RAGE were detected by ELISA kit. In addition, Cognitive assessment was performed by the Mini-Mental State Examination (MMSE) and the Montreal Cognitive assessment (MoCA).

**Results:**

The average RNFL thickness in the right eye were decreased in T2DM and T2DM combined with MCI (T2DM-MCI) patients and were positively correlated with MoCA and MMSE scores. The serum levels of IL-18, CML and RAGE in T2DM and T2DM-MCI increased significantly (p<0.05) and were negative correlated with MoCA and MMSE scores. The level of irisin in T2DM and T2DM-MCI decreased significantly (p<0.05) and were positively correlated with MoCA and MMSE scores. The area under the ROC curve of T2DM-MCI predicted by the average RNFL thickness in the right eye, CML and RAGE were 0.853, 0.874 and 0.815. The diagnostic efficacy of the combination of average RNFL thickness in the right eye, CML, and RAGE for the diagnosis of T2DM-MCI was 0.969.

**Conclusion:**

The average RNFL thickness in the right eye, CML and RAGE have possible diagnostic value in T2DM-MCI patients.

## Introduction

Diabetes Mellitus (DM) and cognitive impairment are prevalent conditions among older individuals. DM, a metabolic disorder with multiple underlying causes, is characterized by chronic hyperglycemia and stands as one of the fastest-growing diseases. Its global prevalence is expected to affect around 693 million adults by 2045, making it a significant health concern (1). In 2019, statistics indicated that approximately 1 in 11 individuals had DM, with a global total of 463 million cases, out of which about 90% were classified as Type 2 diabetes mellitus (T2DM) (2). DM's complications are diverse, encompassing cardiovascular, ocular, renal, and neuropathic issues, as well as disorders of the immune system.

Cognitive impairment resulting from T2DM has garnered significant attention in recent times. This impairment can be categorized into two main classifications: dementia and mild cognitive impairment (MCI). Research has unequivocally demonstrated a connection between T2DM and cognitive decline. Individuals afflicted with T2DM are inherently predisposed to a heightened risk of developing both MCI and full-fledged dementia when compared to those without diabetes (3). In the advanced stages of dementia, the observable symptoms in patients are conspicuous. However, treatment options remain limited. Consequently, this situation places a substantial burden not only on the affected families but also on society as a whole. Therefore, the imperative of identifying early markers for dementia diagnosis and implementing timely interventions has become more pressing than ever before.

Contemporary research underscores the intricate mechanisms underlying cognitive impairment in DM, encompassing disrupted lipid metabolism, inflammatory responses, mitochondrial dysfunction, and oxidative stress. These cumulative factors culminate in a diminished frequency of neuronal activity, the apoptotic demise of nerve cells, anomalous central nervous system integration, distorted information processing, degenerative necrosis, and the demyelination of nerve cells and fibers (4). Of note, inflammation emerges as a pivotal player in the pathogenesis of cognitive impairment in DM. Consequently, our endeavors have been directed towards the identification of pertinent serological indicators for the diagnosis of T2DM in conjunction with MCI, with a specific focus on the inflammatory dimension.

Currently, MCI can be diagnosed by magnetic resonance imaging, positron emission tomography, cerebrospinal fluid (CSF) tests and neuropsychological assessment. But these tests have the disadvantages of being costly, invasive, insensitive and specific. However, to our knowledge, there are no reports of observed changes in RNFL thickness in T2DM-MCI patients. Exploring potential markers for MCI in T2DM is critical to better comprehend its pathogenesis and monitor disease progression. Considering that the potential relation between RNFL degeneration and cognitive deterioration in T2DM remains undiscovered, it would be valuable and fascinating to investigate whether RNFL thickness could become a potential indicator for MCI in T2DM. The aim of this study was to first determine thickness of RNFL in T2DM and T2DM-MCI patients, and to further clarify the correlation between RNFL thickness, concentrations of serum IL18, irisin, CML, RAGEs and cognitive function in T2DM-MCI patients.

## Materials and methods

### Patients

A total of 108 patients diagnosed with T2DM, who were hospitalized in the Endocrinology Department of the Second Hospital of Shandong University between June 2021 and December 2021, were selected for this study. Among them, there were 60 T2DM patients without MCI (34 males and 26 females), and 48 T2DM-MCI patients (34 males and 26 females). Additionally, 23 individuals were recruited as a normal control group (8 males and 15 females). The study was approved by the Ethics Committee of the Second Hospital of Shandong University. Every participant was required to provide a written consent for this study to allow investigators to measure their clinical status. This study has been engaged in the clinical trial registration at http://www.chictr.org.cn/showproj.aspx?proj=186818. And the clinical trial registration number is ChiCTR2300070232.

### Inclusion and exclusion criteria

Inclusion criteria were: 1. Control group: Fasting blood glucose <6.1mmol/l and blood glucose <7.8mmol/l after 2h of Oral Glucose Tolerance Test (OGTT). A score of ≥26 on the Montreal Cognitive assessment (MoCA) and ≥27 on the Mini-Mental State Examination (MMSE). Other acute and chronic diseases were excluded. 2.T2DM group: According to the 2019 WHO diagnostic criteria for diabetes: (1) DM is typically characterized by the symptoms of “three excesses and one deficit (excessive drinking, excessive urination, excessive eating and weight loss)”, random blood glucose level ≥11.1mmol/l. (2) Fasting blood glucose level ≥ 7 mmol/l. (3) OGTT: blood glucose ≥11.1mmol/l after 2h of OGTT. (4) Glycated hemoglobin (HbA1c) ≥6.5%. Patients are diagnosed with diabetes when any of these criteria are met within 2 consecutive days, while excluding type 1 diabetes, gestational diabetes and specific types of diabetes. And the MoCA score ≥ 26 and MMSE score ≥ 27. 3.T2DM-MCI group: Meets the 2019 WHO criteria for diagnosis of DM. And MoCA score is of 18-26 and MMSE score is of 21-27. 4. In all three groups above: best corrected visual acuity ≥ 0.2. Diopter requirements: Spherical lenses -3.00S∼+3.00S, cylindrical lenses -3.00C∼+3.00C, refractive error ≤ 2D in both eyes. IOP <21mmHg in both eyes on all three measurements.

Subjects with any of the following conditions were excluded: 1.Diseases affecting the optic nerve such as glaucoma, retinal nerve fiber layer disorders, high myopia and a history of eye surgery within six months. 2. Other neurodegenerative diseases that may cause changes of the retinal nerve fiber layer, such as multiple sclerosis. 3. Refractive interstitial clouding is evident. 4. Heart failure, malignant hypertension, acute myocardial infarction, stroke, history of renal failure, severe cardiac arrhythmias. 5.Presence of other neurological disorders that cause cognitive impairment, such as brain tumors, Parkinson's, encephalitis, epilepsy, traumatic brain injury, etc. 6.Presence of other conditions that can cause cognitive impairment, such as hypothyroidism, severe anemia, folic acid and vitamin B12 deficiency, syphilis, AIDS, alcohol and drug abuse, etc. 7.Are using medications that affect cognition, such as sedatives, anti-anxiety medications, hypnotic medications, etc. 8.Those who are unable to cooperate with the inspection.

### Research methodology

#### Data collection

(1) General information: Inquire about and record the name, gender, age, hospitalization number, duration of T2DM, education level, previous medical history, family history of the individuals. (2) Basic measurements: height, weight, Body Mass Index (BMI), systolic blood pressure, diastolic blood pressure. (3) Biochemical parameters: fasting blood glucose, HbA1c, triglycerides, total cholesterol, high density liptein cholesterol (HDL-C), low-density lipoprotein cholesterol (LDL-C), estimated glomerular filtration rate (eGFR), liver function, uric acid.

#### Cognitive function

Cognitive function assessments were conducted by the same physician for all participants, utilizing both the MMSE and the MoCA. The cognitive evaluations for the enrolled patients were carried out on the same day as the blood sample collection.

#### OCT image acquisition

All participants underwent bilateral OCT examinations conducted by experienced ophthalmologists from the Second Hospital of Shandong University, and the doctors were unaware of the grouping of participants. The peripapillary region surrounding the optic disk was segmented into four parts, being, superior, inferior, temporal and nasal sectors. The global RNFL thickness represents the average thickness of these four sectors.

#### Testing of serological indicators

Morning fasting blood samples were taken from all participants following a fasting period of 8-10 hours. These samples were then placed in specialized inert separator gel procoagulation tubes. Subsequently, they underwent centrifugation at a speed of 3000 revolutions per minute for a duration of 10 minutes. The resulting supernatant, meticulously isolated, was then stored within a temperature-controlled environment at -80°C until the time of analysis.

#### Data analysis

The required sample size for this study was calculated using the PASS 15 software. The specific parameter settings are as follows: The One-Way Analysis of Variance model was selected, and the significance level α was set to 0.05, and the power of the test (1-β) was set to 0.90. The Number of Groups was set to 3, and Hypothesized Means were set as 110 80 70. The Standard Deviation of Subjects was set to 15. After calculation, the study requires a sample size of 15 individuals. Considering a dropout rate of 20%, each group should include a minimum of 18 patients. All experimental data were statistically analyzed using SPSS 25.0. Means ± standard deviations (x±s) were used for measures meeting normal distribution, and one-way ANOVA was used for data comparison between the three groups. Independent samples *t*-test and paired *t*-test were used for data comparison between the two groups. Non-normally distributed measures were expressed using median and interquartile spacing, and data were compared between the three groups using non-parametric tests and between the two groups using the Mann-Whitney rank sum test. Frequency, percentages or composition ratios were used to describe the count data, and the chi-square test was used for comparison between groups. Correlation analysis was performed using Pearson correlation analysis for normally distributed measures and Spearman for non-normally distributed measures. Binary logistic regression was applied to analyze the independent risk factors and protective factors for MCI in patients with T2DM. Analysis of the diagnostic efficacy of specificity indicators for MCI in patients with T2DM using ROC curves. A *p*-value of less than 0.05 was considered statistically significant.

## Results

The study encompassed a cohort of 23 normal controls, 60 T2DM patients, and 48 T2DM-MCI patients. However, for various reasons such as suboptimal image quality, imaging artefacts, or the presence of macular oedema in OCT imaging, a total of 5 normal controls, 19 T2DM patients, and 12 T2DM-MCI patients were excluded from the subsequent analysis. Therefore, the final dataset subjected to analysis consisted of 18 normal controls, 41 T2DM patients, and 36 T2DM-MCI patients.

### Clinical information for each group

As depicted in **Table 1**, notable statistically significant differences were observed among the three groups with respect to gender, years of education, diastolic blood pressure, blood glucose, HbA1c, and BMI (p<0.05). Conversely, when comparing the T2DM group with the T2DM-MCI group, no statistically significant disparities were evident in terms of gender, years of education, diastolic blood pressure, blood glucose, HbA1c, and BMI (p<0.05).

**Table 1:** Basic information of the study subjects.

	Control (n=18)	T2DM (n=41)	T2DM-MCI (n=36)	*p*
Gender (Male)	7(38.9%)	27(65.9%)^a^	23(63.9%)^a^	**0.016**
Age (year)	53.78±12.82	56.93±9.22	59.78±7.68	0.088
Diabetes history (year)	–	9.90±7.41	10.04±6.86	0.932
years of education (year)	14.72±5.69	9.34±3.64^a^	8.78±2.26^a^	**<0.001**
Systolic pressure (mmHg)	129.22±3.62	134.46±15.89	131.86±15.37	0.408
Diastolic blood pressure (mmHg)	77.33±3.91	85.78±10.94^a^	83.36±8.82^a^	**0.007**
BMI (kg/m^2^)	23.41±0.63	26.10±3.54^a^	26.25±3.45^a^	**0.005**
Blood glucose (mmol/l)	5.48±0.34	9.56±2.84^a^	8.92±2.78^a^	**<0.001**
HbA1c(;%)	5.46±0.27	8.60±1.51^a^	8.75±2.13^a^	**<0.001**
ALT(U/L)	22.00(13.50-27.00)	18.00(12.00-24.50)	18.00(12.00-26.00)	0.755
AST(U/L)	17.00(15.50-22.50)	18.00(15.00-23.00)	18.50(13.00-22.25)	0.619
Triglycerides (mmol/l)	1.13±0.58	1.70±1.64	1.56±0.97	0.277
Total cholesterol (mmol/l)	4.51±0.89	4.79±1.67	4.20±1.38	0.202
HDL-L (mmol/l)	1.31±0.31	1.23±0.55	1.17±0.38	0.575
LDL-C (mmol/l)	2.43±0.76	2.84±0.90	2.52±1.02	0.186
Urine (mmol/l)	5.61(4.83-5.98)	5.60(4.90-6.60)	5.80(4.90-6.90)	0.526
Creatinine (μmol/l)	70.78±7.45	72.52±16.68	75.41±23.64	0.661
Uric acid (μmol/l)	271.97±60.25	302.47±74.87	291.85±82.07	0.385
eGFR (ml/min/1.73m^2^)	91.90±13.64	95.39±19.00	89.77±21.40	0.447

a versus control, p<0.05.

b versus T2DM, p<0.05. Significant P values (<0.05) are indicated in bold.

### RNFL thickness, serum marker concentrations in male and female

As presented in **Table 2** and **Table 3**, there was no difference in RNFL thickness, serological indicator concentrations between male and female (*p*>0.05).

**Table 2:** RNFL thickness in males and females.

	Male	Female	*p*
RNFL thickness in left eye(μm)			
Superior	133.33±18.68	141.58±48.01	0.244
Inferior	124.88±18.22	129.55±22.12	0.264
Nasal	65.93±14.37	71.39±14.94	**0.077**
Temporal	78.12±19.11	81.79±33.75	0.501
Global	101.04±11.25	106.24±23.44	0.151
RNFL thickness in right eye(μm)			
Superior	127.65±19.22	129.79±15.99	0.572
Inferior	124.28±23.03	132.24±21.00	0.091
Nasal	69.77±13.57	74.32±21.22	0.206
Temporal	81.55±21.57	88.66±29.96	0.213
Global	101.02±13.29	106.45±14.25	0.061

Significant P values (<0.05) are indicated in bold.

**Table 3:** Serological indicator concentrations in males and females.

	Male	Female	*p*
Irisin(ng/ml)	34.97(9.34-87.34)	27.85(9.79-66.56)	0.682
CML(pg/ml)	175.32(98.99-324.76)	204.02(83.52-285.28)	0.805
IL-18(pg/ml)	46.74(16.60-171.19)	60.07(16.66-183.89)	0.571
RAGE(pg/ml)	436.30(239.00-703.31)	439.07(266.46-616.01)	0.878

### RNFL thickness of the study subjects

**Figure 1:**
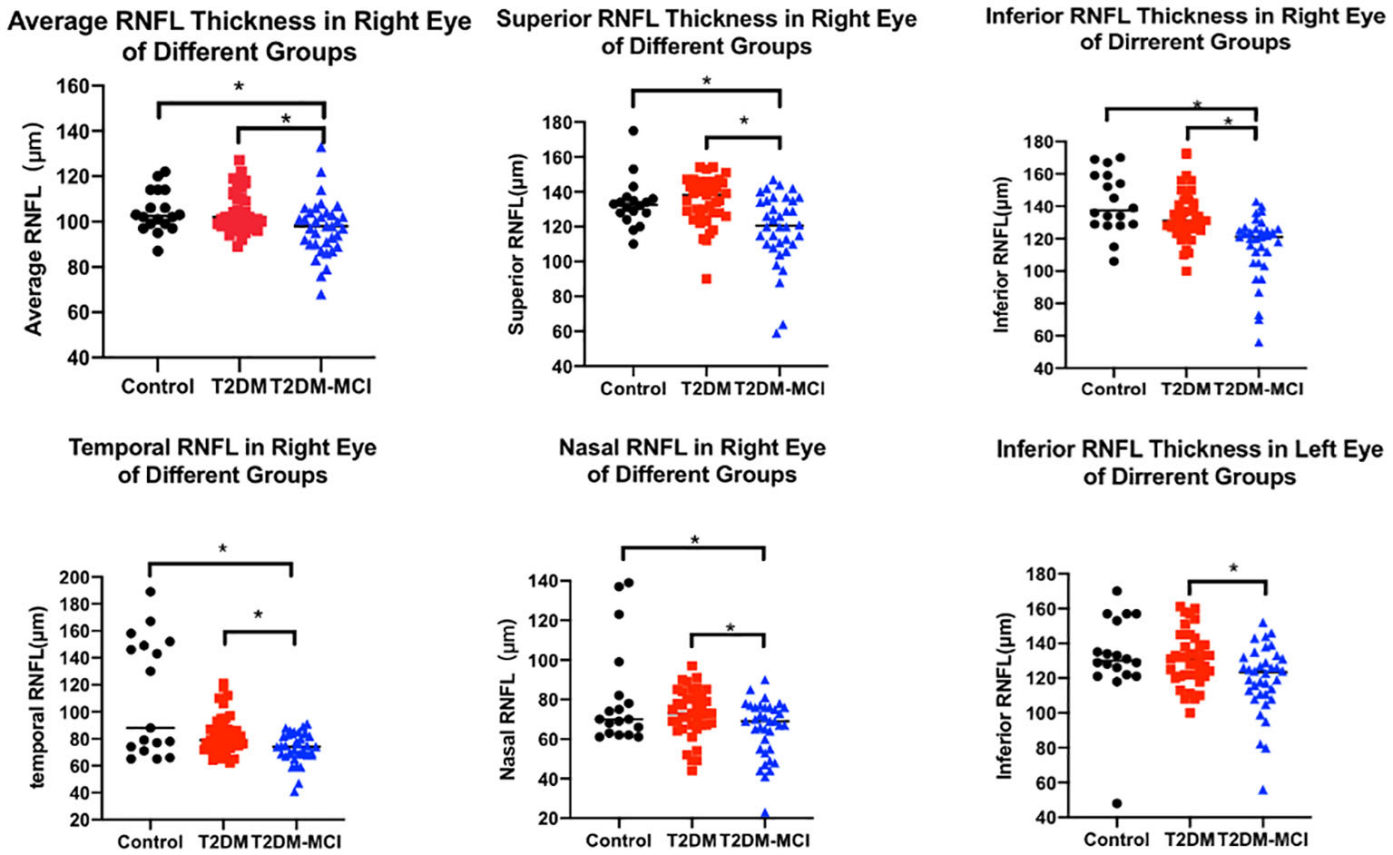
RNFL thickness in different groups. The scatter plots accompanied by the mean ± SD present the RNFL thickness in control, T2DM and T2DM-MCI groups. *p<0.05.

As illustrated in **Table 4** and **Figure 1**, the average RNFL thickness of four quadrants and RNFL thickness in each quadrant of the right eye exhibited notable disparities among the three participant groups (p<0.05). When compared to the control group, differences in RNFL thickness within each quadrant and the average RNFL thickness in T2DM patients were statistically insignificant for the left eye (all p>0.05). However, these measurements were notably reduced in the temporal quadrant and the average RNFL thickness in the right eye (all p<0.05). Furthermore, the T2DM-MCI group displayed reduced average RNFL values and RNFL thickness in each quadrant of the right eye (all p<0.05) in comparison to controls. When comparing the T2DM group with the T2DM-MCI group, the latter exhibited significant thinning in the lower left eye, each quadrant of the right eye, and the average RNFL thickness (all p<0.05).

**Table 4:** RNFL thickness in different groups.

	Control	T2DM	T2DM -MCI	*p*
RNFL thickness in left eye (μm)				
Superior	139.78±11.26	137.17±14.98	134.44±52.06	0.855
Inferior	131.61±25.94	131.17±15.07	119.28±19.59^b^	**0.015**
Nasal	71.33±16.13	68.07±13.26	66.56±15.83	0.538
Temporal	75.06±15.30	80.05±12.60	81.33±38.58	0.699
Global	104.50±9.14	104.37±8.98	101.00±25.70	0.652
RNFL thickness in right eye (μm)				
Superior	133.33±14.08	134.68±13.40	119.06±20.35^a,b^	**<0.001**
Inferior	141.83±18.62	133.83±15.56	113.03±23.29^a,b^	**<0.001**
Nasal	81.06±25.79	72.95±12.09	65.31±14.32^a,b^	**0.004**
Temporal	111.59±43.43	82.90±14.45^a^	73.33±11.15^a,b^	**<0.001**
Global	115.56±15.94	106.34±7.65^a^	93.42±11.74^a,b^	**<0.001**

a versus control, p<0.05.

b versus T2DM, p<0.05. Significant P values (<0.05) are indicated in bold.

### Serum index concentration of each group

**Table 5** presents the concentrations of serological indicators within each group. Statistically significant differences were observed in the concentrations of IL-18, irisin, CML, and RAGE among the groups encompassing T2DM, T2DM-MCI, and the control subjects. When compared to the normal control group, IL-18, CML, and RAGE exhibited significant increments in both the T2DM and T2DM-MCI groups, while the levels of irisin showed a noteworthy reduction. Furthermore, in comparison to the T2DM group, the concentrations of IL-18, CML, and RAGE in the T2DM-MCI group experienced a substantial increase, concomitant with a marked decrease in irisin levels.

**Table 5:** Serum index concentrations of the three groups.

	Control	T2DM	T2DM-MCI	*p*
IL-18(pg/ml)	13.86(11.30-15.58)	32.00(19.83-157.62)^a^	136.36(80.95-407.87)^a,b^	**<0.001**
Irisin(ng/ml)	116.22(77.00-159.01)	32.81(9.07-81.67)^a^	15.69(4.71-31.61)^a,b^	**<0.001**
CML(pg/ml)	16.02(5.08-35.30)	180.09(132.51-237.05)^a^	322.43(277.33-583.87)^a,b^	**<0.001**
RAGE(pg/ml)	193.79(109.40-263.86)	433.02(284.18-566.38)^a^	703.29(475.96-1117.90)^a,b^	**<0.001**

a versus control, p<0.05.

b versus T2DM, p<0.05. Significant P values (<0.05) are indicated in bold.

### Correlation analysis of Moca, MMSE and other indicators

Correlation analysis of clinical features, RNFL thickness, IL-18, irisin, CML, RAGE with MoCA and MMSE was performed in **Table 6**. Average RNFL thickness, age, years of education, HbA1c, total cholesterol, LDL-C, irisin, CML, IL-18, and RAGE were significantly associated with MoCA and MMSE.

**Table 6:** Correlation between Moca, MMSE and other variables.

	MoCA		MMSE	
	r	*p*	r	*p*
inferior quadrant in left eye (μm)	-0.157	0.129	-0.374	**<0.001**
superior quadrant in left eye (μm)	0.163	0.115	0.113	0.275
nasal quadrant in left eye (μm)	-0.074	0.475	-0.132	0.203
temporal quadrant in left eye (μm)	-0.251	**0.014**	-0.417	**<0.001**
average RNFL thickness in left eye (μm)	-0.146	0.158	-0.340	**0.001**
inferior quadrant in right eye (μm)	0.447	**<0.001**	0.346	**0.001**
superior quadrant in right eye (μm)	0.399	**<0.001**	0.284	**0.005**
nasal quadrant in right eye (μm)	0.209	**0.042**	0.145	0.161
temporal quadrant in right eye (μm)	0.365	**<0.001**	0.292	**0.004**
average RNFL thickness in right eye (μm)	0.517	**<0.001**	0.396	**<0.001**
Age (year)	-0.283	**0.005**	-0.331	**0.001**
Diabetes history (year)	0.011	0.912	-0.047	0.684
years of education (year)	0.385	**<0.001**	0.396	**<0.001**
Systolic pressure (mmHg)	0.026	0.805	-0.111	0.285
Diastolic blood pressure (mmHg)	0.056	0.590	-0.029	0.781
BMI (kg/m^2^)	-0.037	0.723	-0.046	0.661
Blood glucose (mmol/l)	-0.031	0.769	-0.025	0.812
HbA1c(;%)	-0.272	**0.009**	-0.283	**0.006**
Triglycerides (mmol/l)	0.131	0.206	0.157	0.128
Total cholesterol (mmol/l)	0.305	**0.003**	0.217	**0.035**
HDL-L (mmol/l)	0.030	0.776	0.010	0.924
LDL-L (mmol/l)	0.218	**0.034**	0.262	**0.010**
eGFR (ml/min/1.73m^2^)	0.025	0.816	-0.030	0.779
Irisin (pg/ml)	0.724	**<0.001**	0.456	**<0.001**
CML(pg/ml)	-0.773	**<0.001**	-0.475	**<0.001**
IL-18(pg/ml)	-0.775	**<0.001**	-0.544	**<0.001**
RAGE(pg/ml)	-0.735	**<0.001**	-0.352	**<0.001**

Significant P values (<0.05) are indicated in bold.

### Logistic regression analysis of MCI in T2DM patients

We employed two distinct models in our study to investigate the associations between RNFL thickness, IL-18, CML, RAGE, and irisin levels, and their relationship to the presence of MCI in patients with T2DM (refer to **Table 7**). Given the known correlations between cognitive function and variables such as age, years of education, HbA1c, total cholesterol, and LDL-C, we initially addressed logistic regression analysis in model 1. This allowed us to correct for the influence of age and years of education, thus enabling a focused exploration of the links between MCI and the average RNFL, IL-18, irisin, CML, and RAGE in T2DM patients. Our findings revealed significant associations. Specifically, we observed that the average RNFL thickness in the right eye was inversely related to the presence of T2DM with MCI (OR=0.699, CI: 0.532-0.920, p=0.011). Moreover, CML (OR=1.011, 95% CI: 1.001-1.021, p=0.030) and RAGE (OR=1.007, 95% CI: 1.001-1.013, p=0.017) levels in the right eye were positively associated with T2DM patients having MCI. Specifically, higher average RNFL thickness in the right eye appeared to be a protective factor, while elevated levels of CML and RAGE emerged were as risk factors for MCI in this context. To account for potential confounding effects stemming from other variables, we conducted further adjustments in model 2, incorporating HbA1c, total cholesterol, and LDL-C. Our results from this analysis continued to highlight significant relationships. Notably, the average RNFL thickness in the right eye remained a protective factor against the presence of T2DM with MCI (OR=0.695, 95% CI: 0.521-0.928, p=0.013). Conversely, CML(OR=1.012, 95% CI: 1.001-1.022, p=0.037) and RAGE (OR=1.007, 95% CI: 1.002-1.012, p=0.007) levels remained as identified risk factors associated with MCI in T2DM patients, even after accounting for the effects of HbA1c, total cholesterol, and LDL-C. Upon examination, the goodness-of-fit for both models were satisfactory.

**Table 7:** Logistic regression analysis of the associations of NPDR with IL-17A, IL-22, and irisin in T2DM patients.

Characteristics	Model 1		Model 2	
	OR (95%CI)	*p*	OR (95%CI)	*p*
Age (year)	0.959(0.854-1.077)	0.481	0.943(0.826-1.076)	0.380
years of education (year)	0.960(0.728-1.266)	0.772	1.018(0.752-1.378)	0.907
HbA1c (%)	–	–	1.468(0.736-2.929)	0.276
LDL-C (mmol/L)	–	–	0.678(0.212-2.161)	0.511
Average RNFL thickness in left eye (μm)	1.161(0.967-1.394)	0.110	1.154(0.938-1.418)	0.175
Average RNFL thickness in right eye (μm)	0.699(0.532-0.920)	**0.011**	0.695(0.521-0.928)	**0.013**
IL-18 (pg/ml)	1.002(0.993-1.012)	0.598	1.002(0.992-1.013)	0.675
CML (pg/ml)	1.011(1.001-1.021)	**0.030**	1.012(1.001-1.022)	**0.037**
RAGE (pg/ml)	1.007(1.001-1.013)	**0.017**	1.007(1.002-1.012)	**0.007**
Irisin (pg/ml)	1.019(0.952-1.092)	0.585	1.022(0.951-1.100)	0.550

Significant P values (<0.05) are indicated in bold.

### Right eye RNFL thickness, CML, RAGE predict MCI in T2DM patients

**Figure 2:**
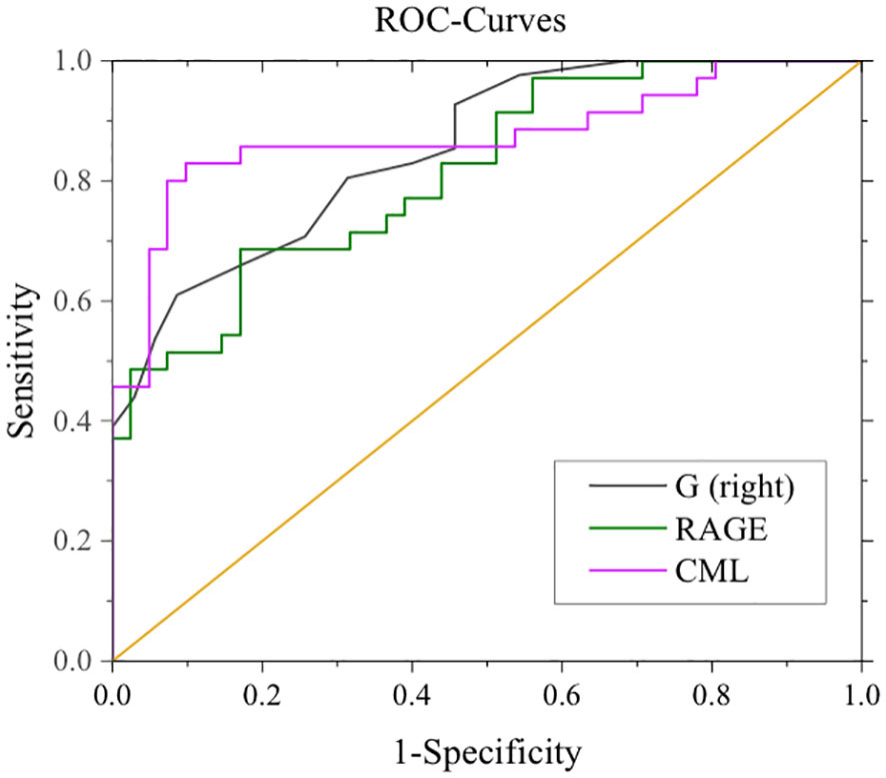
ROC curves for mean RNFL in the right eye, CML, RAGE, and MCI. G (right eye): AUC 0.853, sensitivity 91.7%, specificity 61.0%. CML: AUC 0.874, sensitivity: 82.9%, specificity: 90.2%. RAGE: AUC 0.815, Sensitivity 68.6%, Specificity 82.9%.

**Figure 3:**
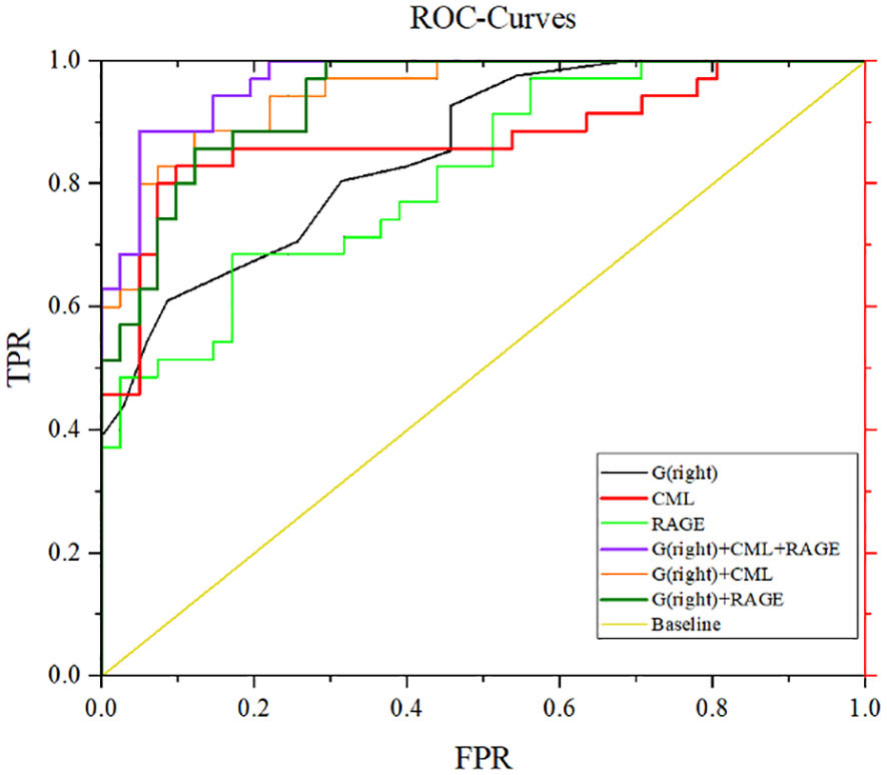
ROC curves for mean RNFL thickness, CML, and RAGE diagnosis in the right eye. G (right eye) + CML: AUC 0.948, sensitivity 88.6%, specificity 87.8%. G(right eye)+RAGE:AUC 0.939, sensitivity 82.9%, specificity 87.8%. G(right eye)+CML+RAGE: AUC 0.969, sensitivity 88.6%, specificity 95.1%.

After adjustment for confounding factors, it was found that average thickness of RNFL, CML and rage in the right eye were still related to cognitive function. In **Figure 2**, ROC curve was used to analyze the diagnostic efficacy of these three indicators for MCI in T2DM. The area under the ROC curve of average RNFL thickness in the right eye for MCI in T2DM was 0.853, the sensitivity was 91.7%, and the specificity was 61.0%. The area under the ROC curve of CML was 0.874, the sensitivity was 82.9%, and the specificity was 90.2%. The area under the ROC curve of RAGE was 0.815, the sensitivity was 68.6%, and the specificity was 82.9%. In order to further explore the significance of the combined diagnosis of OCT and serological indexes, firstly, the average RNFL thickness and CML of the right eye were used as diagnostic indexes to observe whether the combined diagnosis of the two could improve the diagnostic efficacy of MCI in patients with T2DM. The diagnostic efficacy of the combined diagnosis of the two was 0.948 (sensitivity 88.6%, specificity 95.1%), which was significantly higher than that of RNFL thickness and CML of the right eye. The diagnostic efficacy was significantly higher than that of RNFL thickness and CML in the right eye. Next, the diagnostic efficacy of the combined diagnosis of average RNFL thickness and RAGE in the right eye was observed, and it was found that the diagnostic efficacy of the combined diagnosis of the two (0.939) was higher than that of RNFL thickness and RAGE in the right eye. Finally, the diagnostic efficacy of the combined diagnosis of RNFL thickness, CML, and RAGE in the right eye was found to be significantly higher (0.969). (**Table 8**, **Figure 3**).

**Table 8:** Diagnostic efficacy of right eye RNFL thickness, CML, and RAGE in the ROC curve for MCI in patients with T2DM.

Diagnostic indicators	Diagnostic efficacy	Sensitivity	Specificity
G(right)	0.853	91.7%	61.0%
CML	0.874	82.9%	90.2%
RAGE	0.815	68.6%	82.9%
G(right)+CML	0.948	88.6%	87.8%
G(right)+RAGE	0.939	82.9%	87.8%
G(right)+CML+RAGE	0.969	88.6%	95.1%

## Discussion

Under physiological conditions, the curves depicting RNFL thickness exhibit a distinctive "bimodal" configuration. This intriguing pattern manifests with the superior and inferior RNFL thickness surpassing that of the nasal and temporal regions. Notably, the superior and inferior nerve fibers exhibit the highest density, rendering the RNFL in these two quadrants particularly vulnerable to initial damage. In our current investigation, the RNFL thickness profiles of the three patient groups conspicuously displayed this intriguing "bimodal" attribute. Furthermore, a comprehensive retrospective and meta-analytic inquiry unveiled compelling findings. It discerned a significant reduction in the average RNFL thickness among patients afflicted with Alzheimer's Disease (AD) when compared to their cognitively intact counterparts. In AD patients, all quadrants exhibited a pronounced thinning of the RNFL. Intriguingly, a discernible trend towards a thinner RNFL was also observable among patients with MCI. However, it is noteworthy that the extent of this thinning in MCI patients did not reach statistical significance (5). In a community-centered investigation encompassing a substantial cohort of individuals in good health, those devoid of substantial cognitive impairment but exhibiting thinner RNFL measurements at the study's onset were observed to face an elevated risk of experiencing diminished performance on subsequent cognitive assessments (6). The investigation encompassed measurements of peripapillary RNFL thickness using OCT in both AD and MCI patients. The findings revealed a noteworthy pattern of thinning in the RNFL, with particular prominence in the upper quadrant, observed in both AD and MCI patients. Moreover, AD patients exhibited thinning not only in the upper quadrant but also in the lower quadrant when compared to the control group (7). In the initial stages of AD, a discernible pattern emerges involving a selective reduction in the RNFL, which is predominantly localized in the upper quadrant. However, as AD advances, the degenerative process extends beyond the upper quadrant, and also affects the lower quadrant of the RNFL. From an anatomical standpoint, the axons originating from the upper quadrant of the retina project through the parietal segment of the optic radiations, eventually connecting to the cuneate gyrus within the primary visual cortex. Conversely, axons originating from the lower quadrant of the retina project to the lingual gyrus. Notably, the density of senile plaques and neurofibrillary tangles in the cuneate gyrus is greater than that in the lingual gyrus, and this difference may be the reason for the major RNFL lacunae in the middle and upper quadrant of AD (8). Post-mortem examinations of individuals with AD, juxtaposed with control subjects, have unequivocally revealed optic nerve degeneration and a notable decline in retinal ganglion cells (RGC) (9). This observed phenomenon may be attributable to the accumulation of amyloid deposits within the retina, a consequence of cognitive impairment, which subsequently leads to the depletion of RGCs. Collectively, the findings from these aforementioned studies underscore the correlation between the thinning of the RNFL and the progression of Alzheimer's Disease, reaffirming the potential utility of RNFL thickness as an indicative marker for AD development.

In the early stages of neurodegeneration in DM, a cascade of events unfolds, primarily affecting the function and integrity of retinal neurons. This multifaceted process encompasses several key phenomena, including the dysfunction and degeneration of retinal neurons, apoptosis of retinal nerve cells induced by hyperglycemia, heightened neurofilament phosphorylation, increased reactivity of glial cells due to metabolic stress, activation of microglia, and perturbations in the regulation of glutamate levels. Notably, these initial alterations predominantly target the inner retina and manifest as a reduction in the thickness of the RNFL and the loss of ganglion cell bodies (10). Srinivasan et al. reported a notable reduction in the overall thickness of the retina, along with discernible thinning of the RNFL specifically within the pars plana and periocular regions among patients diagnosed with T2DM (11). Among patients afflicted with T2DM who do not exhibit concurrent diabetic retinopathy, evidence of retinal neurodegeneration is discernible. It is noteworthy that defects in the RNFL are a common occurrence in T2DM, frequently manifesting within the upper half of the retina (12). The aforementioned study initially highlighted a thinning of the RNFL in patients diagnosed with T2DM. The outcomes of our present investigation effectively reinforce this established observation. Nevertheless, it is worth noting that, in our current study, we identified a distinct pattern of RNFL thinning in T2DM patients, specifically manifesting in the temporal region. It is important to acknowledge that this particular finding may be attributed to the inclusion of some patients with early-stage glaucoma that may have been challenging to diagnose accurately. In glaucoma patients, RNFL thinning predominantly occurs in the nasotemporal region. To gain a more comprehensive understanding of this phenomenon, future research endeavors would benefit from an expanded sample size and extended participant follow-up, aimed at providing further clarification on this matter. Crucially, our study represents the inaugural effort to investigate alterations in RNFL thickness among individuals with both T2DM and MCI. After meticulously accounting for the potential confounder of gender, we ascertained that RNFL thickness was indeed reduced in patients presenting both T2DM and MCI, in comparison to those with T2DM alone. This distinctive change was predominantly evident in the right eye. It is plausible that this observed phenomenon is underpinned by the established correlation between RNFL thickness and hippocampal volume, particularly as MCI patients tend to exhibit initial atrophy in the right hippocampal region (13). Hence, it is evident that alterations in RNFL thickness among patients presenting both T2DM and MCI initially manifest on the right side. Subsequent investigations could incorporate imaging data to provide further substantiation for this assertion. Remarkably, our findings unveil a noteworthy decline in RNFL thickness among patients diagnosed with AD and those grappling with MCI, in contrast to individuals in a normal cognitive state. Notably, we observed a positive correlation between RNFL thickness and cognitive performance scores, specifically the MoCA and MMSE. This correlation implies a potential simultaneous occurrence of retinal nerve fiber degeneration and central nervous system (CNS) degeneration (14).

Neuroinflammation serves as a natural response within the CNS to both external and internal damage, functioning as a protective mechanism for the brain. However, it's important to note that an excessive or prolonged inflammatory response can be detrimental to the CNS. One key player in this intricate inflammatory cascade is IL-18, a pro-inflammatory cytokine categorized within the IL-1 family. Initially recognized as "IFN-γ-inducible factor," IL-18 has garnered substantial attention due to its involvement in a spectrum of infectious, metabolic, and inflammatory conditions. This includes but is not limited to instances such as influenza virus infections, atherosclerosis, diabetes, chronic obstructive pulmonary disease, and Crohn's disease, where there exists ample evidence of IL-18's pivotal roll (15). Chronic cytokine-mediated inflammation is a prominent feature in T2DM, underscoring the pivotal role of the inflammatory mechanism in the onset and progression of T2DM (16). Notably, our study unveiled that T2DM patients exhibited significantly elevated serum levels of Interleukin-18 (IL-18) compared to the control group (17). As a proinflammatory factor, IL-18 assumes a central role in both the inception and progression of T2DM, a finding consistent with previous research results. The upsurge in circulating IL-18 levels may signify a state of chronic inflammation within the brain, and its overexpression could potentially trigger tau protein phosphorylation, consequently contributing to neurodegeneration in Alzheimer's Disease (AD) patients (18). In post-mortem brain samples from AD patients, IL-18 was detected within microglia, astrocytes, and neurons. These brain alterations are known to contribute to cognitive decline (19). However, there has been a lack of research examining serum IL-18 changes in individuals with both T2DM and cognitive impairment, and how these changes correlate with cognitive function. Our study is pioneering in revealing that IL-18 levels were notably higher in T2DM-MCI patients compared to those with T2DM alone and individuals in the normal control group. Moreover, IL-18 exhibited a negative correlation with MoCA and MMSE scores, shedding new light on the intricate relationship between IL-18 and cognitive function in this specific population.

Irisin, an adipokine secreted by muscle tissue, comprises fibronectin type III domain-containing 5 (FNDC5). Enzymatic cleavage of the carboxyl terminus of FNDC5 by protein hydrolases releases irisin, and this protein is expressed not only in skeletal muscle but also in various other tissues (20). Irisin exhibits the potential to exert multiple beneficial effects on glucose metabolism and insulin sensitivity. It achieves this by stimulating energy expenditure, enhancing glucose uptake, promoting glycogenolysis, and concurrently reducing gluconeogenesis, lipogenesis, and lipid accumulation (21). The majority of clinical investigations have consistently revealed lower irisin levels in individuals with prediabetes or T2DM when compared to their non-diabetic counterparts. This phenomenon is likely attributed to diminished FNDC5 synthesis, resulting in reduced irisin secretion within the muscle tissue of obese individuals and those afflicted with T2DM (22). Current research has consistently demonstrated that irisin exerts a protective influence on T2DM by enhancing insulin sensitivity. Our own investigation corroborates these findings, revealing a significant reduction in serum irisin levels among T2DM patients in comparison to the control group. Furthermore, studies have unveiled the presence of FNDC5 and irisin in both murine and human brains, with particular localization within the hippocampus (23). Irisin, notably, plays a pivotal role in modulating the release of inflammatory factors by astrocytes and promoting neuronal survival (24). Additionally, irisin has been implicated in the transcriptional regulation of Brain-Derived Neurotrophic Factor (BDNF), neurotrophins, and myocytokines. Its deficiency or reduction has been linked to neurodegenerative conditions that have the potential to impact cognitive function. This occurs through the promotion of hippocampal neurogenesis, reduction of oxidative stress, enhancement of glucose metabolism, and augmentation of synaptic plasticity (25). Lourenco and colleagues made a significant discovery, noting reduced levels of FNDC5 in the brains of human Alzheimer's Disease, cerebrospinal fluid, and AD mouse models. Their research further demonstrated that elevating FNDC5 levels within the brain or peripherally had a mitigating effect on synaptic and memory impairments in AD mouse models (26). However, there has been a limited number of studies exploring the role of irisin in patients with T2DM and MCI. In light of this, we have revisited the association between serum irisin levels and T2DM-MCI patients. Our findings reaffirm the significance of irisin as a protective factor in this specific population. We observed that serum irisin levels in T2DM-MCI patients were notably lower in comparison to those with T2DM alone and individuals in the normal control group. Furthermore, we identified a significant negative correlation between irisin levels and MoCA as well as MMSE scores. These results provide further substantiation for the protective role of irisin in T2DM patients with MCI.

Advanced AGEs are isomeric compounds originating from the non-enzymatic reaction between glucose or other sugar derivatives and proteins or lipids. These compounds have been identified in excess of 20 distinct forms within blood, tissues, and various food sources. Notable examples include CML, CEL, pyrrolizidine acid, pentosidine, and methylglyoxal lysine dimer (27). RAGE, an acronym for Receptor for Advanced Glycation End Products, belongs to the immunoglobulin superfamily of cell surface molecules. Positioned within the major histocompatibility complex class III site, RAGE serves as a receptor for ligands known as AGEs. This interaction primarily occurs through its V-type region, which serves as a critical site facilitating intracellular signal transduction. Recent investigations have shed light on the connection between heightened AGE formation within a metabolically perturbed environment and its consequences on cellular responsiveness to insulin. Specifically, this process impacts insulin sensitivity, insulin secretion, and the overall insulin signaling cascade. Ultimately, these disruptions contribute to the development of insulin resistance, which is a pivotal factor in the onset of DM (28). AGEs not only serve as biomarkers indicating hyperglycemia, pro-inflammatory states, or oxidative conditions but also actively participate in the pathogenesis of complications associated with DM. These multifaceted roles are largely mediated through their interactions with the Receptor for RAGE (29). Indyk et al. made a noteworthy discovery by documenting significant elevations in serum AGEs and sRAGE levels among diabetic patients. This finding further reinforces the compelling link between AGEs and their respective receptors in the context of diabetes development (30). In the diabetic state, there is an upsurge in endogenous AGE production, concomitant with the upregulation of RAGE and the downregulation of the AGE scavenger receptor AGR1. This cascade of events potentially contributes to a reduction in the clearance of AGEs, amplifying their impact within the system (31). Research has unveiled compelling evidence indicating that for each 100ng/ml rise in CML levels, there is an associated 35% increase in the risk of developing diabetes (32). Indeed, all the aforementioned studies have consistently demonstrated elevated serum levels of CML and the RAGE in individuals diagnosed with T2DM. Our own investigation corroborates these findings, underscoring significantly higher levels of CML and RAGE among T2DM patients when compared to their normal counterparts. Moreover, our current study has reported that exposure to AGEs leads to an upregulation of APP expression, both *in vitro* and *in vivo* experiments. This elevated APP expression subsequently results in increased levels of β-amyloid, suggesting a potential significant role for AGEs as a risk factor in the pathogenesis of AD (33). Activation of RAGE exacerbates not only Aβ production and aggregation but also the formation of neurofibrillary tangles (NFTs). Additionally, RAGE activation disrupts synaptic transmission and neuronal function, thereby promoting the onset and progression of AD. Inhibition of RAGE has been shown to mitigate Aβ-induced damage in neuronal cells and cerebral vasculature (34). Presently, all available research consistently underscores the pivotal role of the AGEs-RAGE pathways in the pathogenesis of AD. However, there is a notable absence of relevant studies investigating the involvement of AGEs-RAGE in individuals with T2DM combined with MCI. In this investigation, we have made a groundbreaking discovery, demonstrating for the first time that levels of CML and the RAGE were markedly elevated in patients diagnosed with T2DM combined with MCI, in comparison to those with T2DM alone and individuals in the normal control group. Furthermore, we have established significant negative correlations between serum CML and RAGE concentrations and MoCA as well as MMSE scores. To further substantiate the role of the CML-RAGE pathway in the pathogenesis of T2DM combined with MCI, future animal experiments will be conducted at subsequent stages of this research.

In this study, we made several noteworthy findings. Firstly, we observed elevated serum levels of IL-18, CML, and the RAGE in patients with T2DM. These elevations were positively correlated with MoCA and MMSE scores. Furthermore, we noted significantly lower levels of irisin and a reduction in the average RNFL thickness in the right eye among T2DM patients. Irisin and RNFL measurements also exhibited positive correlations with MoCA and MMSE scores. To ensure the robustness of our findings, we conducted a correction analysis, factoring in potential confounders such as age, years of education, HbA1c, and LDL-C levels. Even after accounting for these variables, the associations between CML, RAGE, RNFL thickness in the right eye, and cognitive impairment remained statistically significant. Our ROC curve analysis further underscored the diagnostic potential of these indicators for MCI in T2DM patients. The average RNFL thickness in the right eye exhibited an impressive area under the curve (AUC) of 0.853, along with a sensitivity of 91.7%, indicating its strong potential as a diagnostic tool for MCI in T2DM patients. Similarly, CML demonstrated an AUC of 0.874 with a specificity of 90.2%, suggesting its effectiveness in minimizing false negatives in MCI diagnosis. RAGE, with an AUC of 0.815 and a specificity of 82.9%, also displayed potential for reducing false negatives in MCI diagnosis. These findings collectively introduce novel avenues for identifying MCI in T2DM patients. However, it is imperative that future research endeavors encompass more centers and larger sample sizes to further validate the utility of these three indicators as a basis for diagnosing MCI in individuals with T2DM.

In comparison to utilizing serological indices or OCT examinations in isolation, the combined diagnostic approach demonstrates superior capability in distinguishing MCI from healthy individuals. Within this study, we identified three key markers for diagnosing MCI in patients with T2DM through regression and correlation analyses. Our findings strongly advocate for the adoption of a combined diagnostic model encompassing average RNFL thickness, CML, and the RAGE in the right eye. This amalgamated model offers enhanced diagnostic efficacy, thus proposing its potential as a supplementary tool in clinical diagnostics. Nevertheless, the study population for this research is derived from a single center, and being a cross-sectional study, it has inherent limitations. Subsequent investigations could involve multi-center, prospective studies for further exploration.

## Conclusion

In summary, our study reveals important correlations between cognitive function and specific biomarkers. Notably, the average RNFL thickness in the right eye exhibited a positive correlation with cognitive function. Conversely, serum CML and RAGE levels displayed negative correlations with cognitive function. These findings suggest the potential diagnostic utility of these three indicators in identifying MCI in individuals with T2DM.

## Data availability statement

The raw data supporting the conclusions of this article will be made available by the authors, without undue reservation.

## Ethics statement

The studies involving humans were approved by the ethics committee of the Second Hospital of Shandong University. The studies were conducted in accordance with the local legislation and institutional requirements. The participants provided their written informed consent to participate in this study.

## Author contributions

RL: Data curation, Writing – original draft. FZ: Data curation, Writing – original draft. LL: Formal analysis, Writing – original draft. PX: Investigation, Writing – original draft. YM: Methodology, Software, Writing – original draft. XZ: Funding acquisition, Project administration, Writing – review & editing. SC: Funding acquisition, Project administration, Resources, Writing – review & editing.
