# Corrigendum: Correlation of mild cognitive impairment with the thickness of retinal nerve fiber layer and serum indicators in type 2 diabetic patients

**DOI:** 10.3389/fendo.2024.1380375

**Published:** 2024-04-25

**Authors:** Renshi Li, Fengjie Zheng, Peichen Xu, Li Lv, Yapeng Mu, Xianghua Zhuang, Shihong Chen

**Affiliations:** Department of Endocrinology and Metabolism, Multidisciplinary Innovation Center for Nephrology of the Second Hospital of Shandong University, The Second Hospital of Shandong University, Ji’nan, Shandong, China

**Keywords:** type 2 diabetes mellitus, the thickness of retinal nerve fiber layer, interleukin-18, irisin, carboxymethyl lysine, receptors for AGEs, mild cognitive impairment

In the published article, there was an error in the author affiliations. The first author’s affiliation should be “Department of Endocrinology and Metabolism, Multidisciplinary Innovation Center for Nephrology of The Second Hospital of Shandong University, The Second Hospital of Shandong University, Ji’nan, Shandong Province, China”, instead of “Emergency department, Affiliated Hospital of Jiangsu University, Zhenjiang, Jiangsu Province, China”.

The authors apologize for this error and state that this does not change the scientific conclusions of the article in any way. The original article has been updated.

